# Strong interaction of CpcL with photosystem I cores induced in heterocysts of *Anabaena* sp. PCC 7120

**DOI:** 10.17912/micropub.biology.001183

**Published:** 2024-05-27

**Authors:** Takehiro Suzuki, Haruya Ogawa, Naoshi Dohmae, Jian-Ren Shen, Shigeki Ehira, Ryo Nagao

**Affiliations:** 1 Biomolecular Characterization Unit, RIKEN Center for Sustainable Resource Science, Saitama 351-0198, Japan; 2 Research Institute for Interdisciplinary Science and Graduate School of Natural Science and Technology, Okayama University, Okayama 700-8530, Japan; 3 Department of Biological Sciences, Graduate School of Science, Tokyo Metropolitan University, Tokyo 192-0397, Japan; 4 Faculty of Agriculture, Shizuoka University, Shizuoka 422-8529, Japan

## Abstract

Phycobilisomes (PBSs) are photosynthetic light-harvesting antennae and appear to be loosely bound to photosystem I (PSI). We previously found unique protein bands in each PSI fraction in heterocysts of *Anabaena* sp. PCC 7120 by two-dimensional blue native/SDS-PAGE; however, the protein bands have not been identified. Here we analyzed the protein bands by mass spectrometry, which were identified as CpcL, one of the components in PBSs. As different composition and organization of *Anabaena* PSI-PBS supercomplexes were observed, the expression and binding properties of PBSs including CpcL to PSIs in this cyanobacterium may be diversified in response to its living environments.

**Figure 1. Identification of CpcL in the PSI tetramer and dimer fractions from heterocysts of Anabaena sp. PCC 7120:**
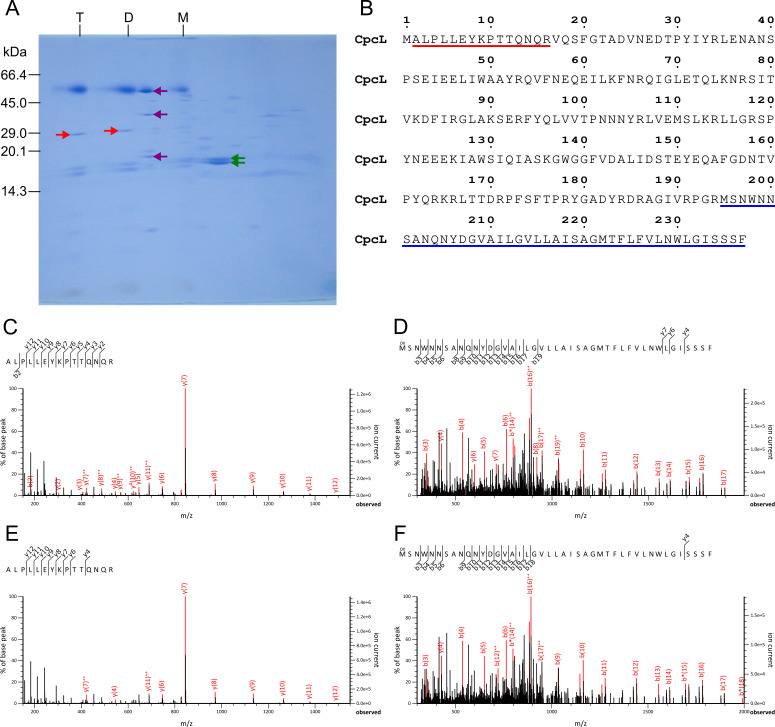
( **A** ) Two-dimensional BN/SDS-PAGE of the heterocyst thylakoids. T, PSI tetramer; D, PSI dimer; M, PSI monomer. Protein bands labeled as red arrows were analyzed by mass spectrometry. Bands labeled as purple and green arrows can be assigned to ATP synthase and PBS proteins, respectively, according to Chang et al. (2015) and Srivastava et al. (2021). This figure was modified from Nagao et al. (2022), Copyright Elsevier. ( **B** ) Amino acid sequence of CpcL. Red and blue underlines indicate N and C-terminal polypeptides, respectively, of CpcL detected by MS/MS analyses ( **C–F** ). ( **C, D** ) MS/MS spectra of ALPLLEYKPTTQNQR (m/z = 591.3284; z = 3+; MASCOT score = 56; Expect value = 6.2e−6) ( **C** ) and MSNWNNSANQNYDGVAILGVLLAISAGMTFLFVLNWLGISSSF (m/z = 1163.5813; z = 4+; MASCOT score = 70; Expect value = 2.6e−7) ( **D** ) within the band labeled as a red arrow in the PSI tetramer fraction in panel **A** . ( **E, F** ) MS/MS spectra of ALPLLEYKPTTQNQR (m/z = 591.3283; z = 3+; MASCOT score = 64; Expect value = 1.0e−6) ( **E** ) and MSNWNNSANQNYDGVAILGVLLAISAGMTFLFVLNWLGISSSF (m/z = 1163.5829; z = 4+; MASCOT score = 76; Expect value = 7.8e−8) ( **F** ) within the band labeled as a red arrow in the PSI dimer fraction in panel **A** .

## Description

Light-harvesting antennae have been diversified in photosynthetic organisms (Blankenship 2021) and can be grouped into membrane proteins and water-soluble proteins. Phycobilisomes (PBSs) are water-soluble pigment-protein complexes and consist of linker proteins and pigment-binding phycobiliproteins (MacColl 1998; Adir 2005; Watanabe and Ikeuchi 2013; Rast et al. 2019; Adir et al. 2020) . PBSs are primarily bound to photosystem II of cyanobacteria and red algae as revealed by cryo-electron microscopy (Rast et al. 2019; You et al. 2023) . However, it is unclear how PBSs interact with photosystem I (PSI) to catalyze light-induced electron transfer from plastocyanin or cytochrome *c*_6_ to ferredoxin (Golbeck 1992; Brettel and Leibl 2001; Fromme et al. 2001; Nelson and Junge 2015) .

Watanabe et al. purified PSI-tetramer-PBS supercomplexes from the cyanobacterium *Anabaena* sp. PCC 7120 (hereafter referred to as *Anabaena* ) grown under normal conditions (Watanabe et al. 2014) , whereas we have purified PSI-dimer-PBS and PSI-monomer-PBS supercomplexes from *Anabaena* grown under iron-deficient conditions (Shimizu et al. 2023) . The two studies detected CpcL in the supercomplexes, which is one of the linker proteins in PBSs and was denoted by Watanabe et al. (2014). CpcL, previously known as CpcG3 in *Anabaena* , is one of the rod-core linker proteins (Bryant et al. 1991) . It contains an N-terminal linker domain and a distinctive hydrophobic C-terminal domain (Kondo et al. 2007; Watanabe et al. 2014; Hirose et al. 2017; Guo et al. 2024) . In contrast, CpcL was also called CpcG2 in the cyanobacterium *Synechocystis* sp. PCC 6803 and its characteristic protein structure has been determined by cryo-electron microscopy (Zheng et al. 2023) .

It is known that CpcL of *Anabaena* was highly expressed in heterocysts (Watanabe et al. 2014) , which are formed under nitrogen-starvation conditions (Wolk et al. 1994; Magnuson and Cardona 2016) . We recently showed that by two-dimensional blue-native (BN)/SDS-PAGE, an apparent protein band in a molecular-weight range of 20.1–29.0 kDa was observed in the PSI fractions obtained from the heterocyst thylakoids of *Anabaena* , but this band was not observed in any PSI fractions from the vegetative thylakoids of *Anabaena* (Nagao et al. 2022) . The protein band is presumed to be CpcL based on its apparent molecular weight; however, it has not yet been definitively identified.

The bands presumed to be CpcL in the two-dimensional BN/SDS-PAGE of the PSI tetramer and dimer fractions from the heterocyst thylakoids (labeled as red arrows in Figure 1A ) were analyzed by mass spectrometry. The results showed that the N- and C-terminal polypeptides of CpcL can be detected clearly in both PSI tetramer and dimer fractions ( Figure 1B –F). Because CpcL was hardly observed in any PSI fractions by two-dimensional BN/SDS-PAGE of vegetative thylakoids from *Anabaena* (Nagao et al. 2021; Nagao et al. 2022) , strong interactions of CpcL with PSI cores appear to be induced in heterocysts.

This study demonstrated that CpcL is tightly bound to at least the PSI tetramer and dimer in the *Anabaena* heterocysts, and it was hardly observed in the PSI monomer fraction ( Figure 1A ). Under iron-deficient growth conditions of *Anabaena* , CpcL was detected from the PSI dimer and monomer fractions but not the tetramer (Shimizu et al. 2023) . Thus, the association of CpcL with PSI oligomers may be changed under different conditions; e.g., iron-deficiency and nitrogen-starvation have induced a different association pattern of CpcL with PSI oligomers. It is interesting to note that any PBS components including CpcL were hardly detected in the PSI fractions from the vegetative thylakoids of *Anabaena* by two-dimensional BN/SDS-PAGE (Watanabe et al. 2011; Nagao et al. 2021; Nagao et al. 2022) , although the PSI tetramer cores together with CpcL were fractionated from the vegetative thylakoids by a milder method of sucrose density gradient centrifugation (Watanabe et al. 2014) . These observations suggest that the tight association of CpcL with PSI cores occurs in *Anabaena* grown under stress conditions, such as iron deficiency and nitrogen starvation. Thus, the expression and binding properties of PBSs including CpcL to PSIs in *Anabaena* may be diversified in response to its living environments.

## Methods

Heterocysts and their thylakoids were prepared as described previously (Nagao et al. 2022) . Two-dimensional BN/SDS-PAGE was carried out as described previously (Nagao et al. 2021; Nagao et al. 2022) . Mass spectrometry was performed according to Nagao et al. (2019) with some modifications. Briefly, the gel slices were digested with trypsin (TPCK-treated; Worthington Biochemical), and the resultant peptides were analyzed using LC-MS/MS consisting of Easy nLC 1000 and Q Exactive (Thermo Fisher Scientific). The acquired data was searched against the database of *Anabaena* sp. PCC 7120 with Mascot Version 2.8 software (Matrix Science).
